# CPD: Test yourself

**Published:** 2010-09

Continuing Professional Development (CPD), also known as Continuing Medical Education (CME), describes courses and activities which help professionals such as health care workers to broaden their knowledge and improve their skills.

Through this **CPD Test yourself section**, we aim to support your continued professional development. We hope that you will use these questions to test your own knowledge and understanding, and that you will discuss them with your colleagues and other members of the eye care team.

These questions have been developed in association with the International Council of Ophthalmology and are based on the style of the ICO Advanced Examination www.icoexams.org/exams/advanced

**Table:** 

**1. Think about the responsibilities of eye care workers for the care and maintenance of equipment. Which of the following statements are true and which are false?**		**True**	**False**
a	It is better to wait no more than one week before reporting a fault with your equipment	□	□
b	Equipment users are responsible for checking the safety of their equipment	□	□
c	Equipment that is being taken out of use (decommissioned) can be disposed of along with other waste	□	□
d	Only junior equipment users are responsible for looking after the equipment they use	□	□
**2. Think about the practical aspects of equipment maintenance and repair. Which of the following statements are true and which are false?**		**True**	**False**
a	Because of their design, it does not matter if foot pedals get wet	□	□
b	A voltage stabiliser or regulator cannot always prevent damage to equipment	□	□
c	You should take bulbs, screwdrivers, and fuses along when going on outreach	□	□
d	There is nothing you can do to prevent fungal growth (mould) forming on optical components	□	□
**3. Think about equipment donations and purchasing equipment**		**True**	**False**
a	When purchasing equipment, order enough spare parts and consumables | for at least two months	□	□
b	You should always accept donated equipment when it is offered	□	□
c	Delays in clearing equipment through customs can end up increasing your costs	□	□
d	Budget and plan for maintenance and repair as soon as the equipment arrives	□	□
**4. Think about training for equipment maintenance and repair**		**True**	**False**
a	highly competitive environment helps equipment users learn better	□	□
b	Formal courses are the only appropriate way of training the equipment maintenance and repair team	□	□
c	The service manual can be very useful in isolated eye units	□	□
d	Even with an in-house workshop, there will always be a need for outsourced maintenance services	□	□
ANSWERS			
**1. a.** False. Faults should be reported immediately, **b.** True **c.** False. Some equipment may contain hazardous materials, **d.** False. All equipment users are responsible and should be trained to use and care for their equipment properly.			
**2. a.** False. Moisture can cause the electrical components to stop working. Always lift off the floor when the floor is being cleaned, **b.** True. Even the best voltage stabiliser cannot prevent all possible damage due to fluctuations in electricity. However, good quality stabilisers will offer better protection, **c.** True. **d.** False. For example, you can place fungicidal (anti-mould) pellets or sachets of silica gel drying agents inside the dust cover, or use dehumidifiers to keep the room dry.			
**3. a.** False. Order enough for one year, or more if spare parts are difficult to obtain, **b.** False. Ensure you have the skills, resources and budget needed to use and care for the equipment. There may also be shipping or customs clearance costs, **c.** True. The shipping company will charge more if they have to store the equipment for a longer time. **d.** False. This might be too late - maintenance and repair services may be unaffordable or not available locally. Rather budget and plan at the time of purchase or before.			
**4. a.** False. A friendly environment is better for teaching equipment users, **b.** False. Work placements (internships) and on-the-job training are just two of the many other ways to train these workers, **c.** True. The service manual contains more detailed information than the user manual. At the time of purchase, try to obtain the service manual from the supplier, **d.** True. Some tasks are too complex for the in-house team.			

